# N-demethylsinomenine metabolite and its prototype sinomenine activate mast cells via MRGPRX2 and aggravate anaphylaxis

**DOI:** 10.3389/fphar.2024.1389761

**Published:** 2024-07-31

**Authors:** Youfa Qin, Yihan Huang, Xiaolan Ji, Ling Gong, Shiqiong Luo, Jiapan Gao, Rui Liu, Tao Zhang

**Affiliations:** ^1^ School of Pharmacy, Health Science Center, Xi’an Jiaotong University, Xi’an, China; ^2^ The Affiliated Dongguan Songshan Lake Central Hospital, Guangdong Medical University, Dongguan, China

**Keywords:** N-demethylsinomenine, sinomenine hydrochloride, MRGPRX2, anaphylaxis, synergistically

## Abstract

Sinomenine hydrochloride (SH) is commonly used in the treatment of rheumatoid arthritis. It activates mast cells and induces anaphylaxis in the clinical setting. Adverse drug reactions can be caused by activation of MAS-associated G protein-coupled receptor X2 (MRGPRX2) on mast cells. Because the ligand binding site of MRGPRX2 is easily contacted in dilute solvents, it can be activated by many opioid drug structures. N-Demethylsinomenine (M-3) has a similar chemical structure to that of the opioid scaffold and is a major metabolite of SH. We sought to clarify whether M-3 induces anaphylaxis synergistically with its prototype in a mouse model. Molecular docking computer simulations suggested a similar binding effect between M-3 and SH. M-3 was chemically synthesized and analyzed by surface plasmon resonance to reveal its affinity for MRGPRX2. Temperature monitoring, *in vivo* hindlimb swelling and exudation test, and *in vitro* mast cell degranulation test were used to explore the mechanism of MRGPrx2 mediated allergic reaction triggered by M-3. Reduced M-3-induced inflammation was evident in MrgprB2 (the ortholog of MRGPRX2) conditional (Cpa3-Cre/MrgprB2flox) knockout (MrgprB2-CKO) mice. Additionally, LAD2 human mast cells with MRGPRX2 knockdown showed reduced degranulation. M-3 activated LAD2 cells synergistically with SH as regulated by GRK2 signaling and IP3R/PLC/PKC/P38 molecular signaling pathways. The results indicate that the M-3 metabolite can activate mast cells synergistically with its prototype SH via MRGPRX2 and aggravate anaphylaxis. These findings provide important insights into drug safety.

## Graphical Abstract

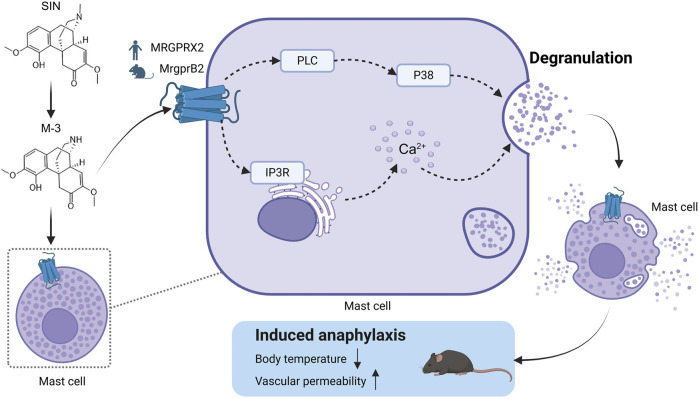

## 1 Introduction

Sinomenine is an active substance extracted used as a traditional Chinese medicine. It is extracted from *Sinomenium acutum* and mediates various kinds of pharmacological actions, with anti-inflammatory, immunosuppressive, antitumor, neuroprotective, and antiarrhythmic efficacy (Li et al., 2023). Zheng Qing Feng Tong Ning is a prescription with sinomenine hydrochloride (SH) as the main ingredient. SH has received approval from the Chinese government for clinical use in treating rheumatoid arthritis (Liu et al., 2018). However, the increased use of sinomenine has been accompanied by the increased incidence of adverse reactions, with clinical anaphylaxis receiving much research attention (Liu et al., 2017). N-Demethylsinomenine (M-3) is a major metabolite of sinomenine (Yao et al., 2007). M-3 has anti-inflammatory effects (Li et al., 2020), attenuates chronic hyperalgesia and inflammatory pain in mice (Zhou et al., 2021), and has pharmacological effects similar to those of SH. However, whether M-3 causes these adverse effects is unknown.

Anaphylaxis is a potentially life-threatening allergic reaction that is not mediated by the immune system. Specific symptoms include rash, redness of the skin, swelling of the skin, nausea, vomiting, and dizziness, among others (Worm et al., 2022). It is reported that among the many positively charged drugs approved by the Food and Drug Administration of the United States (FDA), a receptor called Mas-related G protein-coupled receptor-X2 (MRGPRX2) is expressed in mast cells and can induce allergic reactions. Covers antibiotics, opioids, neuromuscular blockers, antidepressants, and radiographic contrast agents (Mcneil, 2021). MrgprB2 is a homolog of human MrgprX2 (Mcneil et al., 2015). SH induces the release of histamine and other allergic mediators from RBL-2H3 and P815 cells (Huang et al., 2015; Wang et al., 2016). Additionally, MRGPRX2 mediates SH-induced anaphylactoid responses and activating mast cell (Liu et al., 2017). The anti-inflammatory property of M-3 is comparable to that of SH (Li et al., 2020), and its analgesic effect has a faster onset than that of SH (Ou et al., 2018; Zhou et al., 2021). However, whether M-3 contributes to anaphylaxis triggered by SH is unknown. Moreover, various endogenous short peptides, including substance P, PAMP(9-20), and LL-37, and small-molecule drugs, including morphine, sinomenine, and ciprofloxacin, reportedly act as ligands for MRGPRX2. Their regulatory mechanisms differ. How SH activated mast cells differ from those activated by peptides is unknown.

Molecular docking simulations were used to investigate the binding properties of M-3 to MRGPRX2 in this study. In order to explore the mechanism of allergic reaction induced by M-3 *in vivo*, We selected C57BL/6 mice, Mice with conditional knockdown of MRGPRB2 (MRGPRB2-CKO via the CPA3-Cre/MRGPRB2 flox system) and mice with loss of mast cell function by W-SASH C-kit mutant (KitW-sh/W-sh) were studied. We used mast cells from laboratory allergic disease 2 (LAD2) and human embryonic kidney 293 cells, characterized by elevated expression of MRGPrx2, to unravel the targets of M-3 and the mechanisms underlying the *in vitro* activation of anaphylaxis involving calcium mobilization, granule release, and other processes. Additionally, an mRNA sequencing analysis was used to study the differences in gene expression caused by MRGPrx2 activated by small molecules (such as M-3 and SH) and endogenous short peptide Pamp (9-20) (referred to as PAMP-12). The aim of this study was to clarify the mechanism by which metabolites and prototype drugs trigger anaphylaxis through the activation of mast cells by MRGPRX2. The findings provide new insights into drug-induced anaphylaxis.

## 2 Materials and methods

### 2.1 Drugs and reagents

SH with high purity (98%) was provided by Chengdu Pfeidel Biotechnology Co., Ltd. Company (Chengdu, Sichuan, China). Information provided by Sigma-Aldrich, Inc., St. Louis, MO. In the state of Missouri in the United States of America, there is a type of town called Louis. Researchers there discovered a chemical called nitrophenyl N-acetyl-β-D-glucamide. In addition, they studied Evans blue and compound 48/80 (C48/80), a well-known initial secretion stimulator. Saline was purchased from Shandong Qilu Pharmaceutical Co., Ltd. (Jinan, Shandong, China). Fluo-3 AM and Pluronic F-127 cells were purchased from Biotium (Fremont, CA, United States). 2-Aminoethyldiphenylboronate (2-APB), somebody 202,190, GO6983, paroxetine hydrochloride, and YM-254 890 were supplied by MCE (Shanghai, China). Enzyme-linked immunosorbent assay (ELISA) kits were purchased from ExCell Biology, Inc. (Shanghai, China), including human tumor necrosis factor-alpha (TNF-α), interleukin-8 (IL-8) and monocyte chemoattractant protein-1 (MCP-1). Histamine was purchased from Sigma-Aldrich, while histamine · 2HCl (A, A, B, B-D4, 98%) was sourced from Cambridge Isotope Laboratories Ltd. High performance liquid chromatography (HPLC) grade methanol and acetonitrile from Thermo Fisher Scientific (Waltham, MA) were purchased in Cambridge, MA. formic acid, for the liquid chromatography-mass spectrometry (LC-MS) grade, was introduced from Sigma-Aldrich.

### 2.2 Molecular docking assay

In order to study the ligand interactions of receptors, molecular docking experiments were performed with the SURFLEX-DOCKMOde software suite (based on the SYBYL-X2 platform).0; Tripos, St. Louis, Missouri, in the country). The model described earlier provides the basis for the docking model of MRGPRX2. (Cao et al., 2021). The reported MRGPRX2 structure (https://www.rcsb.org/) was used for docking with M-3 and SH.

### 2.3 Surface plasmon resonance

MRGPRX2 protein (Sino Biological, Inc., Beijing, China) at a concentration of 25 μg/mL was fixed on a CM5 sensor chip (Cytiva Sweden AB, Uppsala, Sweden) by capture-coupling. Then, sequential injections of 0, 0 were performed.0064, 0.032, 0. M-3 of 16, 4, 20 and 100 μm, or 0,0.3125, 0.625, 1.25, 2.5, 5, and 10 μmol of thiol compound were combined with running buffer containing 5% dimethyl sulfoxide (DMSO) in phosphate buffered saline (PBS-P). The interaction of MRGPRX2 with bound small molecules was evaluated at 25°C (General Electric Medical Systems, Fairfield, CT). Binding was performed over 180 s and dissociation was performed over 600 s while maintaining a flow rate of 20 μL per second.

### 2.4 Mice

The C57BL/6 strain (6–8 weeks old) was derived from the Xi’an Jiaotong University Laboratory Animal Core (located in Xi’an, China). An experimental model of conditional knockout of MRGPRB2 gene (named MRGPRb2-CKO) was constructed on the basis of C57BL/6 strain, which was verified by PCR. A mast cell deficient mouse strain KitW-sh/W-sh carrying a W-Sash C-kit mutant was provided by the Model Animal Research Center of Nanjing University (Nanjing, Jiangsu). All laboratory rodents were properly housed in the laboratory animal care facilities of Xi’an Jiaotong University and provided with the necessary nutrients and water. All experiments involving consistent procedures on animals were performed by scientific researchers who kept the details of the experiments private.

### 2.5 Ethics statement

This study strictly adhered to the recommendations of the Guide for the Care and Use of Laboratory Animals of the National Institutes of Health. Experimental protocols for the use of mice were approved by the Animal Ethics Committee of Xi’an Jiaotong University (Permit Number: XJTU 2023-157). The study was conducted in accordance with the local legislation and institutional requirements.

### 2.6 Cell lines

The laboratory preparation of MRGPrx2/HEK293 cell line was achieved by PCR and Western blotting. Cells were incubated in DMEM medium containing 10% fetal bovine serum and 100 units of penicillin-streptomycin mixed antibiotics (HyClone, Logan, UT, United States). LAD2 cells provided by researchers Arnold Kirshenbaum and Dean Metcalfe of the National Institutes of Health in Bethesda, Maryland, were propagated in a medium containing STEMPRO-34. The medium incorporates 100 ng/mL of human stem cell growth factor, 100 units of penicillin and streptomycin, 2 mM of L-glutamine, and a nutrient supplement of STEMPRO. The cells were cultured at 37°C in 5% CO2 and maintained a population density of 5 × 10 ^ 5 cells per milliliter.

### 2.7 Intracellular calcium ion (Ca^2+^) mobilization assay

After washing the LAD2 cells with calcium imaging buffer twice, they were incubated with M-3 at concentrations of 0, 10, 50, and 100 μM for 30 min at 37. degree.C.CIB consists of a single component. For the chemical formulation provided, it can be rephrased as a solution containing 0.2 mol per liter of sodium bicarbonate, 125 mmol per liter of sodium chloride, 3 mmol per liter of potassium chloride, and 20 mmol per liter of sucrose.5 mM CaCl2,10 mM HEPES,0. Magnesium salt 6 mmol, sugar 40 mmol, neutral 7.4. Fluo-3 AM 4 µM was fused with Pluronic F-127 0 in the culture medium.1%.The cells were washed twice with CIB. Fill each well with C48/80 mixed with CIB at a concentration of 30 μg/mL.Images were captured using an Eclipse Ti-S fluorescence microscope manufactured by Nikon (Tokyo, Japan) at a magnification of 200. Unless otherwise indicated, agents were injected into the wells within 5 s of the initial imaging, and the response was monitored at a frequency of once every 1 s for 120 consecutive seconds. The reaction progress was followed in real time using an Eclipse Ti-S fluorescence microscope manufactured by Nikon (Tokyo, Japan).

### 2.8 β-hexosaminidase release assay

Tyrode’s solution comprises 120 mM sodium chloride, 4.7 mM KCl, 2.5 mM CaCl2, 1.2 mM MgSO4, 1.2 mM KH2PO4,10 mM HEPES,5.5 mmol of glucose and 5 mmol of bovine serum albumin. LAD2 cells were seeded into 96-well plates at a density of 2 × 10 ^ 4 cells per well, followed by overnight culture. After removal of the medium, M-3, SH, or C48/80 (used as a positive control) were incorporated into the modified Tyrode solution and incubated for 30 min. A 700 mL plate was pelleted at 400 revolutions per minute for 300 s. Cells were lysed in modified Tyrode’s solution containing 0.5%.In the experiment, 1% Triton X-100 solution was used. The β-hexosaminidase was extracted from the supernatant and cell lysate, and its activity of hydrolyzing nitrophenyl N-acetyl-β-D-glucosamine was quantitatively analyzed.1 m citric acid or sodium citrate buffer solution adjusted to pH 4.5). The samples were incubated at 37°C for 90 min, followed by the addition of a stop buffer (0.1 m sodium carbonate/sodium bicarbonate, pH 11.0). The samples were quantitatively analyzed using a spectrophotometer at a wavelength of 405 nm.

### 2.9 Histamine release assay

The samples were analyzed by liquid chromatography-electrospray ionization tandem mass spectrometry (LC-ESI-MS/MS) using an LCMS-8040 mass spectrometer manufactured by Shimadzu Corporation (Kyoto, Japan). Using a Venusil HILIC column (2.1 × 150 mm, 3 μm; Agela Technologies, Tianjin, China) to assess histamine in this system. The histamine was eluted at 0.3 mL/min using an isocratic elution buffer containing 0.1% formic acid and 20 mM ammonium formate (77: 23, v/v).

### 2.10 Measurements of cytokine and protease levels

The concentration of chemokines in cell culture supernatants was quantified using the Human Chemokine Assay Kit according to the manufacturer’s guidelines. After incubating the mixture of cells with M-3, SH, C48/80, or their vectors for up to 6–8 h, the modified Tyrode solution was quantitatively analyzed for TNF-α, IL-8, and MCP-1 using a commercially available ELISA kit according to the second procedure described previously. Follow the manufacturer’s guidelines.

### 2.11 Small interfering RNA (siRNA) transfection of LAD2 cells

Specific siRNAs were used to knock down MRGPRX2, while the siRNA as a control was selected to serve as a Non-treatment group. Derived from DNA drug manufacturers., Ltd. In Shanghai, researchers have successfully constructed an intelligent double-stranded siRNA library, which targets MRGPRX2 and contains non-specific siRNA.Final delivery of siRNA was by Lipofectamine^®^ 2,000-mediated transfection (from Invitrogen, Carlsbad, Calif., United States) at a concentration of 1 μM according to the manufacturer’s guidelines. After 48 h of culture, the expression level of MRGPRX2 in the cells was significantly reduced, followed by quantitative analysis of β-hexosaminidase activity and cytokine secretion. The sequence of the siRNA was as follows: negative control siRNA, forward, 5′-UUC​UCC​GAA​CGU​GUC​ACG​UTT-3′, and reverse 5′-ACG​UGA​CAC​GUU​CGG​AGA​ATT-3’; MRGPRX2 knockdown siRNA, forward, 5′-GUA​CAA​CAG​UGA​AUG​GAA​ATT-3′, and reverse, 5′-UUU​CCA​UUC​ACU​GUU​GUA​CTT-3’.

### 2.12 Synergy determination with SynergyFinder

We seeded LAD2 cells in 96-well plates at 2 × 10^4^ cells/well and they were treated as described next. M-3 and SH lone or in combination were analyzed at the indicated amounts using the aforementioned from β-hexosaminidase. The concentration gradient of M-3 or SH was predetermined according to the efficacy of each drug on mast cell activation. The rate of release rate of β-hexosaminidase from mast cells was analyzed at constant dilution ratios of the two drugs (2 μM, 4 μM, and 8 μM for both M-3 and SH). The rate of release of β-hexosaminidase was assayed as described above after treatment for 30 min. Using the online SynergyFinder software (https://synergyfinder.fimm.fi), the rate of release of β-hexosaminidase was considered as the percentage inhibition ranging from 0 to 1. The drug synergy score was calculated along with the Inhibition Index using response surface modeling and the zero interaction potency (ZIP) calculation method (Ianevski et al., 2022; Zheng et al., 2022). ZIP synergy scores >0 were considered synergistic and are denoted in red, and scores >10 were considered strongly synergistic (Zhang et al., 2022).

### 2.13 Body temperature

The rectal temperature of experimental mice was monitored by FT3400 animal thermometer (produced by Nanjing Kevin Biotechnology Company., Ltd. Nanjing, China). Mice were injected intravenously with 0.200 μL contains 5 mg per milliliter of M-3 substance. By using a bioactivity assessment device, the body temperature of the individual is measured, and a temperature sensor is implanted in the anus to obtain a rectal temperature reading. Temperature checks are taken every 5 min, and the process lasts for an hour.

### 2.14 Hindpaw swelling and extravasation assay

Adult mice (6–8 weeks) were anesthetized with 50 mg/kg pentobarbital sodium intraperitoneally. 15 min after the induction of anesthesia, the rats were given an intravenous injection of 200 μL of 4% Evans Blue. A Vernier caliper was used to measure the thickness of the paws before any injection. After 5 min, one paw was injected with 0.5 mg/ml M-3 or SH or 30 μg/mL C48/80. A reasonable amount of saline was injected into the second paw as a negative control. The paw thickness was measured and recorded after 15 min. The mice were then euthanized with CO_2_. Paw tissues were collected, dried at 65°C, and weighed separately. 1 mL of acetone (7: 3, v/v) solution of acetone (7: 3, v/v) was added and incubated for 6 h at 56°C. The tissues are then ground up, disturbed in an ultrasound machine for 10 min, and centrifuged for 20 min at 3,000 rpm. The supernatants were aliased equally into 96 well plates (200 μL/well), and the optical density (OD) was measured at 620 nm with a microplate reader.

### 2.15 mRNA sequencing analysis

LAD2 cells with a density of 5 × 10^6^ cells/mL were inoculated into 6-well plates. M-3 (50 μmol/L), SH (50 μmol/L), and PAMP-12 (2 μmol/L) was added to the cells for 3 h. After incubation, remove the cell suspension into the corresponding 1.5 mL EP tubes, centrifuge to discard the medium, wash twice with RNase-Free PBS, and centrifuge to obtain the cell precipitate; lysed the cells by adding Trizol solution, and then placed at room temperature for 5–10 min to be used until the cells were completely lysed, and then transferred to a freezing tube to be preserved at −80°C.

The experiment was entrusted to Lianchuan Biotechnology Co., Ltd. for subsequent on-line analysis and basic data analysis. The samples were tested and analyzed for correlation, differentially expressed genes, differentially expressed gene function and pathway annotation and KEGG enrichment analysis. After data acquisition, bioinformatics analysis was performed on the data.

### 2.16 β-hexosaminidase release detection under the inhibitors

LAD2 cells were seeded at a density of 2 × 10^4^ cells/well in a 96-well plate. Then they were treated with 50 μL 2-APB (45 μM), SB202190 (10 μM), and CCT129957 (3 μM), respectively. 30 min later, the cells were added 50 μM M-3additionally. Then the cells were incubated for 30 min. And the β-hexosaminidase release ratio were detected as described in 2.8.

### 2.17 Statistical analysis

The data set is expressed in the form of mean ± SD. Independent-sample t-tests were performed using IBM’s SPSS statistical software in Armonk, New York, to assess the statistical differences between different samples. Differences were considered significant at *p* < .05 (^*^ *p* < .05, ^**^ *p* < .01, and ^***^ *p* < .001, depending on the experiment).

## 3 Results

### 3.1 Molecular docking reveals activation between SH, M-3, and MRGPRX2

**FIGURE 1:**
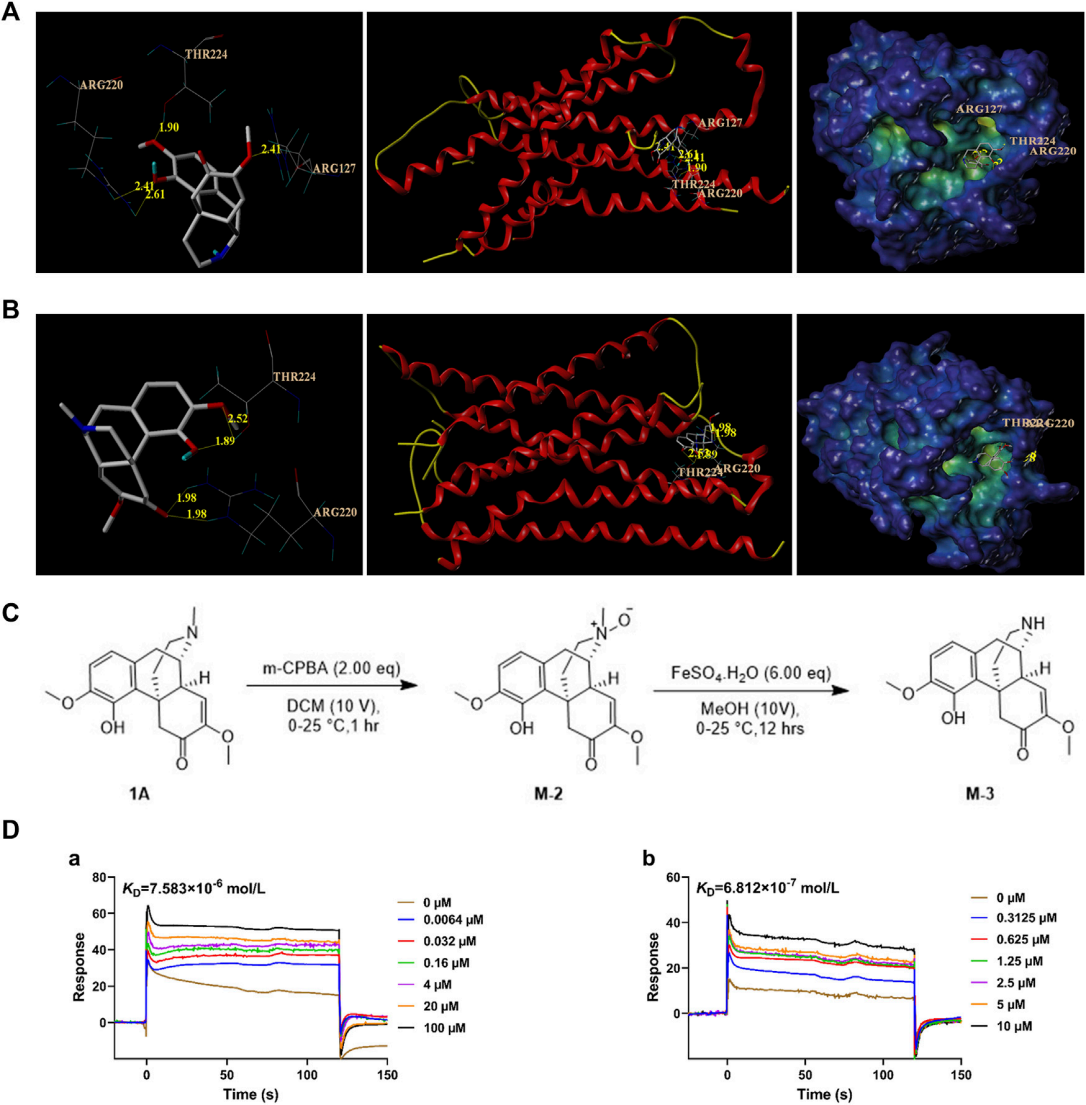
Affinity analysis between M-3 and MRGPRX2. **(A)** Molecular docking model of M-3 with MRGPRX2. **(B)** Molecular docking model of SH with MRGPRX2. **(C)** M-3 synthesis process. **(D)** The binding curves of M-3 (a) and SH (b) on MRGPRX2, and the *K*_D_ values were 7.583 × 10^−6^ mol/L (M-3) and 6.812 × 10^−7^ mol/L, respectively.

As more protein and nucleic acid structures have become available, molecular docking is considered for compound screening on the basis of its increased accuracy (Shoichet et al., 2002). To investigate the differences in the interactions between M-3, SH, and MRGPRX2, molecular docking simulations were performed using the Surflex-Dock Mode of the SYBYL-X 2.0 program package. The simulations investigated ligand–protein interactions between M-3, SH, and MRGPRX2. According to the docking results, M-3 and MRGPRX2 formed hydrogen bonds with ARG220 (2.41 Å and 2.61 Å, respectively), THR224 (1.90 Å), and ARG127 (2.41 Å) (Figure 1A). M-3 bound to MRGPRX2 (4.8309) to form hydrogen bonds. SH and MRGPRX2 formed hydrogen bonds with ARG220 (1.98 Å and 1.98 Å, respectively) and THR224 (1.89 Å and 2.52 Å, respectively) (Figure 1B). Binding of SH to MRGPRX2 (5.1807) to form hydrogen bonds was evident. These results indicate that M-3, similar to SH, has a good binding capacity with MRGPRX2.

### 3.2 Chemical synthesis of M-3

In order to carry out follow-up experimental research, M-3 compound were synthesized by chemical methods (Figure 1C). To a solution of **1A** (Figure 1C) (2.00 g, 6.07 mmol, 1.00 eq) in DCM (20.0 mL) was added to MCPBA (2.62 g, 12.14 mmol, 80% purity, 2.00 eq) portion-wise at 0°C. The mixture was stirred at 0°C–25°C for 1 h. LC-MS showed **1A** was consumed completely. These two reactions were then combined together. The reaction mixture was added NH_3_.H_2_O to pH∼9, followed by extraction with three 50-mL volumes of CHCl_3_. The combined organic layers were washed with three 100-mL volumes of 10% Na_2_SO_3_ solution, dried over Na_2_SO_4_, filtered, and concentrated under reduced pressure to obtain the residue, designated M-2. M-2 (4.19 g, crude) was a brown oil. It was used in the next step without further purification.

M-2 (2.09 g, 6.05 mmol, 1.00 eq) was added portion-wise at 0°C to a solution of FeSO_4_.7H_2_O (10.09 g, 36.31 mmol, 6.00 eq) in 60.0 mL methanol. The mixture was stirred at 0°C for 4 h. LC-MS showed that M-2 was completely consumed, and the desired compound was detected. These two reactions were then combined. The reaction mixture was added EDTA (0.60 M, 59.60 mL, 5.91 eq) at 0°C for 4 h. The mixture then received NH_3_·H_2_O until the pH was approximately 9, and was then extracted with three 100-mL volumes of DCM. The combined organic layers were dried over Na_2_SO_4_, filtered, and concentrated under reduced pressure to obtain a residue designated M-3. M-3 was purified using preparative HPLC using a Phenomenex Luna C18 column (100 × 30 mm × 5 um) with a phase of 10 mM NH_4_HCO_3_ acetonitrile in water). M-3 (76.4 mg, 199.87 µmol, 1.65% yield, 82.5% purity) was obtained as an off-white solid and checked by H-nuclear magnetic resonance and LC-MS.

### 3.3 M-3 has affinity with MRGPRX2

After obtaining the synthesized M-3, SPR was used to study its affinity with MRGPRX2. The results indicated the strong and dose-dependent binding of M-3 to MRGPRX2 (*K*_D_ = 7.583 × 10^−6^ mol/L; Figure 1D-a). The calculated interaction strength between SH and MRGPRX2 was *K*_D_ = 6.812 × 10^−7^ mol/L (Figure 1D-b). The collective findings indicate that both metabolite M-3 and its prototype, SH, can activate MRGPRX2 and may cause hypersensitivity.

### 3.4 M-3 induces calcium mobilization in MRGPRX2/HEK293 cells and degranulation of LAD2 cells

**FIGURE 2:**
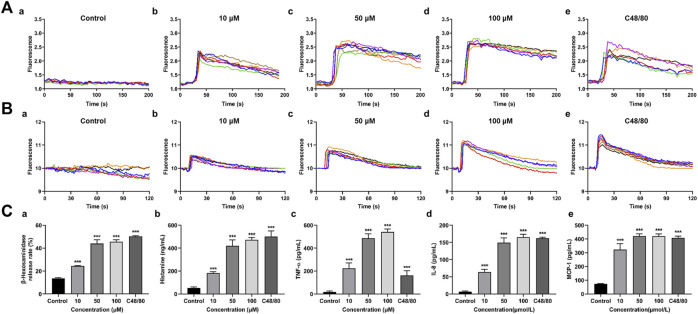
M-3 induces calcium mobilization in MRGPRX2/HEK293 cells and degranulation of LAD2 cells. **(A)** Representative imaging traces of intracellular Ca^2+^ influx treated with M-3 in negative control MRGPRX2/HEK293 cells: (a) Treated with 0 μM M-3, (b) Treated with 10 μM M-3, (c) Treated with 50 μM M-3, (d) Treated with 100 μM M-3, (e) Treated with 30 μg/mL C4880. **(B)** Representative imaging traces of intracellular Ca^2+^ influx treated with M-3 in negative control LAD2 cells: (a) Treated with 0 μM M-3, (b) Treated with 10 μM M-3, (c) Treated with 50 μM M-3, (d) Treated with 100 μM M-3, (e) Treated with 30 μg/mL C4880. **(C)** The degranulation of LAD2 cells triggered by M-3. The β-hexosaminidase (a), histamine (b), TNF-α (c), IL-8 (d) and MCP-1 (e) release in LAD2 cells treated with 10 μM, 50 μM, 100 μM M-3 or 30 μg/mL C48/80 for 30 min.

MRGPRX2 is the human ortholog of mouse Mrgprb2. MRGPRX2/HEK293 cells which were treated with 10, 50, or 100 μM M-3 produced ramarkable dose-dependent increases in intracellular Ca^2+^ mobilization (Figure 2A), similar to the C48/80 positive control. When LAD2 cells were treated in the same manner, M-3 produced a ramarkable dose-dependent increase in intracellular Ca^2+^ migration (Figure 2B). We used LAD2 mast cells as a model to detect changes of the secretion of β-hexosaminidase (a degranulation marker), histamine (mediators of increased vascular permeability) and TNF-α, IL-8, and MCP-1. The treatment of LAD2 cells with 10, 50, or 100 μM M-3 for 30 min and the levels of secreted β-hexosaminidase, histamine, TNF-α, IL-8, and MCP-1 were examined. C48/80 (30 μg/mL) was used as the positive control to verify whether the M-3-induced activation of mast cells promoted degranulation. The three concentrations of M-3 and 30 μg/mL C48/80 promoted the secretion of both β-hexosaminidase (Figure 2C-a) and the anaphylactic mediator histamine (Figure 2C-b). Release of TNF-α, IL-8, and MCP-1 was substantially increased (Figure 2C-c, d, e) in dose-dependent manners.

### 3.5 M-3 induces β-hexosaminidase release from LAD2 cells via MRGPRX2 and can synergize with SH

**FIGURE 3:**
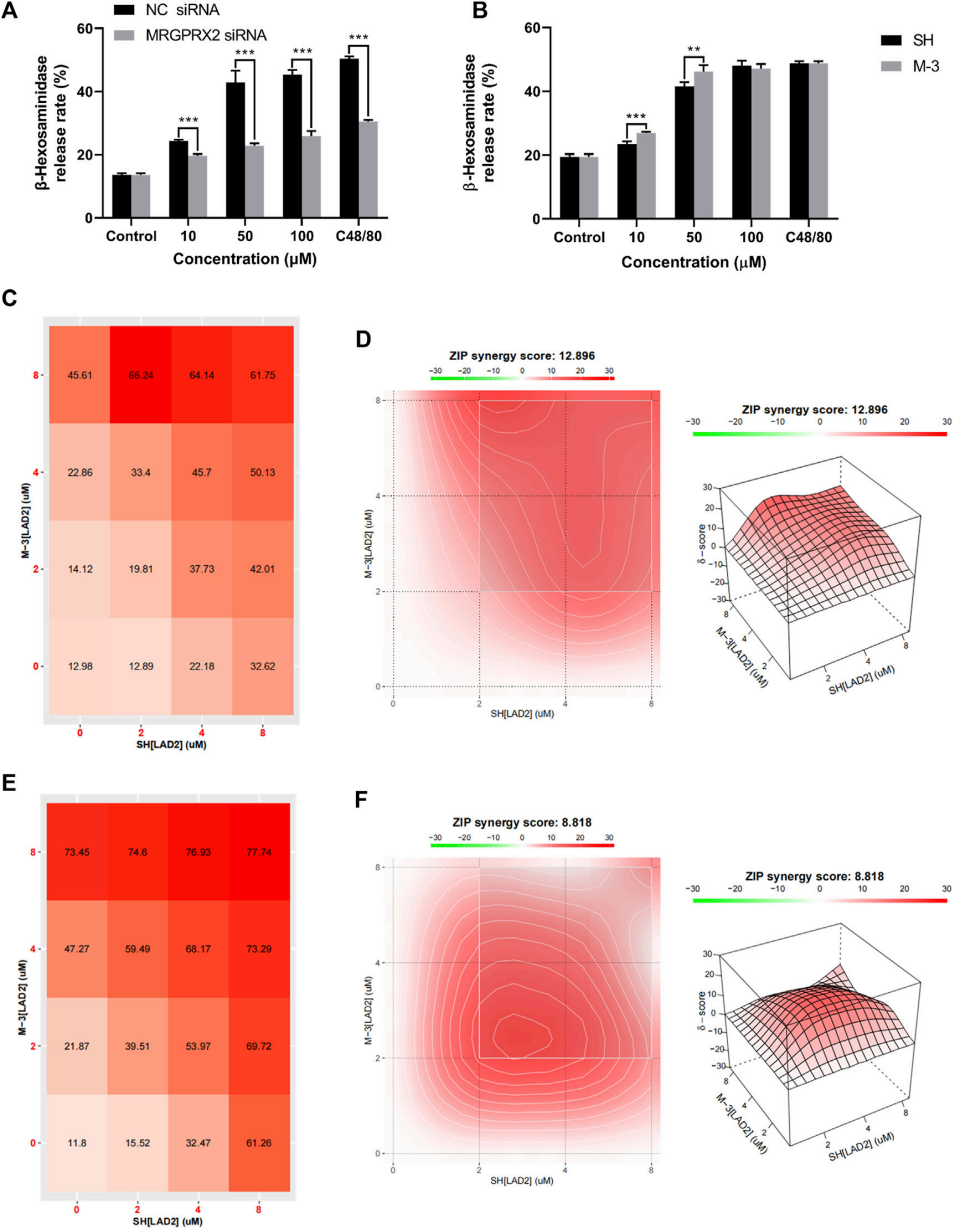
M-3 and SH synergistically activate the release of β-hexosaminidase from LAD2 cells via MRGPRX2. **(A)** The degranulation of MRGPRX2 knockdown LAD2 cells triggered by M-3; **(B)** The degranulation of LAD2 cells triggered by M-3. The β-hexosaminidase release in LAD2 cells treated with 10 μM, 50 μM, 100 μM M-3 or SH for 30 min; **(C)** Rate of β-hexosaminidase release from LAD2 cells under the co-action of M-3 and SH. **(D)** Two-dimensional thermograms and three-dimensional thermograms of β-hexosaminidase release from LAD2 cells under the co-action of M-3 and SH. M-3 and SH act synergistically on LAD2 cells. Cells were treated with the indicated concentrations of M-3 and SH for 30 min and the rate of β-hexosaminidase release was assayed; **(E)** Rate of histamine release from LAD2 cells under the co-action of M-3 and SH. **(F)** Two-dimensional thermograms and three-dimensional thermograms of histamine release from LAD2 cells under the co-action of M-3 and SH. M-3 and SH act synergistically on LAD2 cells. Cells were treated with the indicated concentrations of M-3 and SH for 30 min and the rate of histamine release was assayed. ZIP Synergy scores were calculated using Synergyfinder software. A score of >0 indicates synergy and a score of >10 is considered strong synergy. The gradient in the red area indicates the strength of the synergy. The white rectangle indicates the concentration that contains the highest synergistic region, and the *X* and *Y* axes corresponding to the sides of the white rectangle indicate the concentration at which the drug combination induced the greatest release of β-hexosaminidase.

LAD-2 cells in which MRGPRX2 was knocked down by siRNA treatment were used to validate the interaction between the MRGPRX2 receptors and M-3. Treatment with 0, 10, 50, and 100 μM M-3, or 30 μg/mL C48/80 reduced the release rate of β-hexosaminidase release rate, treated by the same concentration of M-3, respectively (Figure 3A). To compare the differences in mast cell activation by the same concentration of M-3 and SH, we treated LAD2 cells with 10, 50, and 100 μM M-3 and SH, respectively (Figure 3B). M-3 induced release of β-hexosaminidase was greater than SH at 10 and 50 μM. As an active metabolite of SH, M-3 coexists with SH *in vivo*. Accordingly, we investigated in a series of orthogonal experiments whether the two have a synergistic effect in promoting β-hexosaminidase release from mast cells and detected the effect on histamine release at the same time. M-3 and SH were used at different concentration ratios to stimulate the release of β-hexosaminidase and histamine from mast cells. Using SynergyFinder software to calculate The ZIP synergy scores at the detected numerical value, to judge the synergistic effect of M-3 and SH. The ZIP synergistic score revealed a synergistic effect of M-3 and SH on β-hexosaminidase (Figures 3C, D) and histamine (Figures 3E, F) release from LAD2 cells. The findings support the view that the M-3 metabolite can act synergistically with the prototype SH to activate mast cells *in vivo* and aggravate the prototype drug to trigger anaphylactic reactions.

### 3.6 M-3 induces hypothermia

**FIGURE 4:**
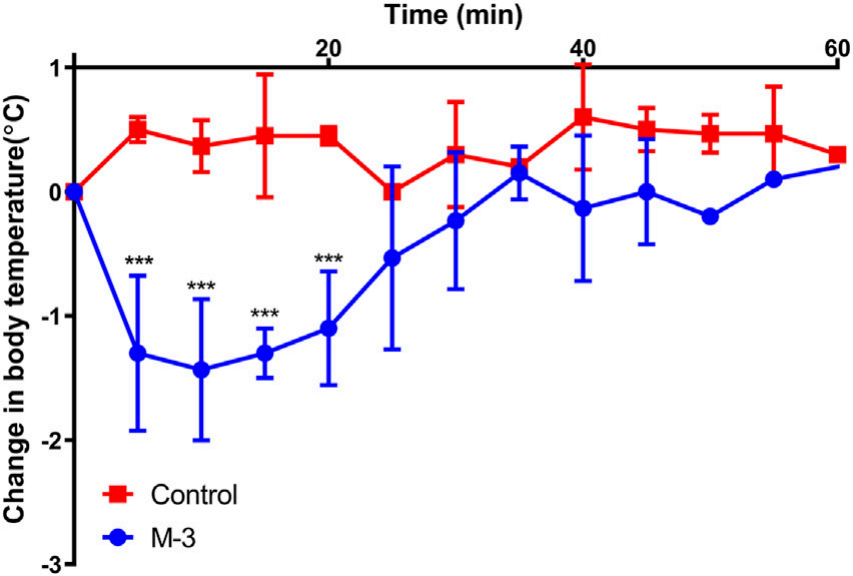
Changes in body temperature after intravenous injection of 10 μg/mL M-3. Data are presented as mean ± standard error of the mean. *n* = 4. ***p < 0.001 vs. Control.

The body temperature of mice decreased rapidly and recovered slowly when anaphylaxis occurred. To investigate whether M-3 causes systemic allergic symptoms in mice, body temperature was monitored for 60 min after intravenous injection of M-3. Within 60 min of injection, the body temperature of the mice dropped sharply, slowly recovered, and eventually returned to normal (Figure 4). The results indicate that M-3 can induce anaphylaxis.

### 3.7 Mrgprb2 is important in M-3 induced paw anaphylaxis

**FIGURE 5:**
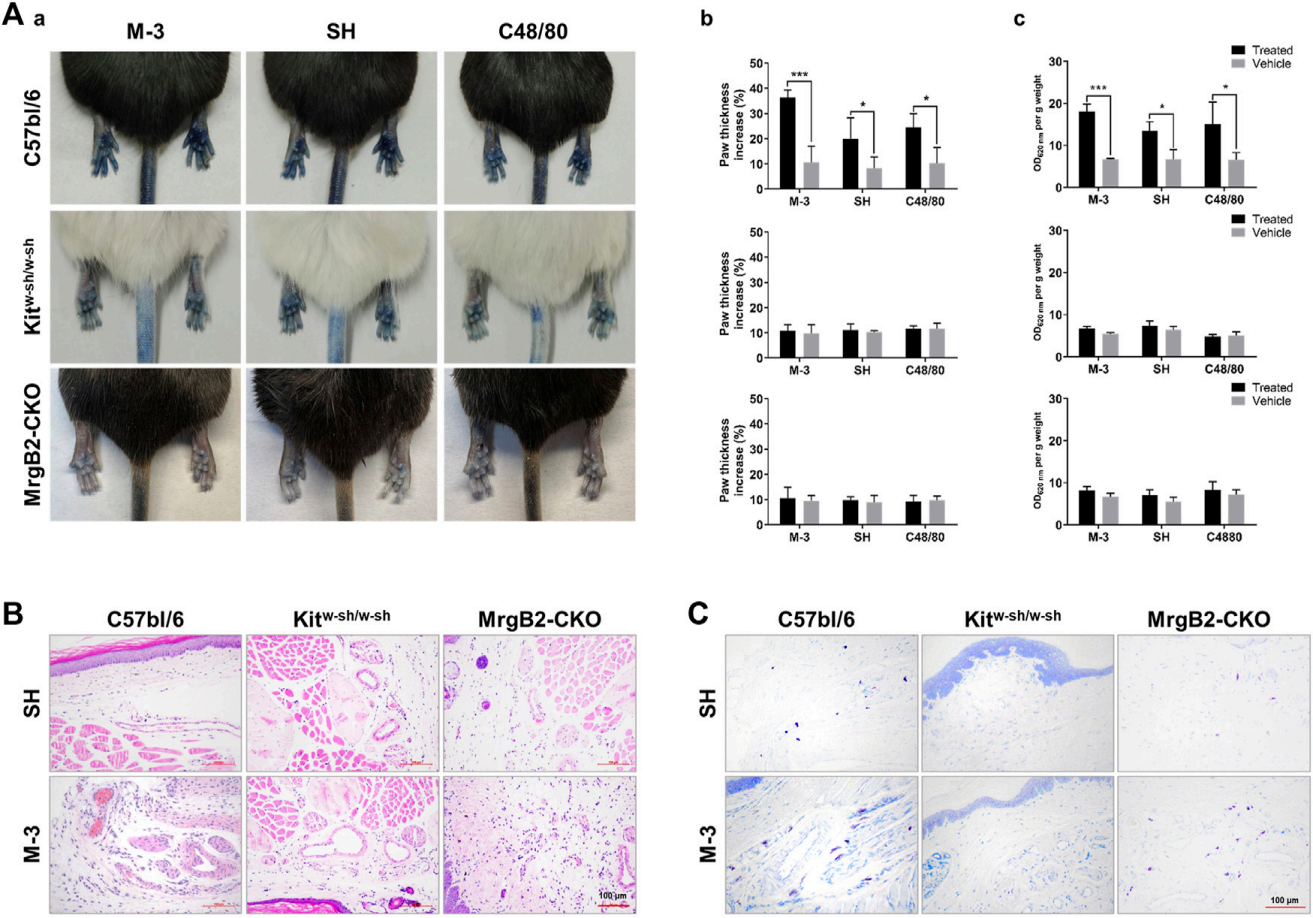
M-3 and SH induce cutaneous flare reactions mediated by MrgprB2 in mast cells in mice. **(A)** Cutaneous flare reactions of mice treated with different concentrations of M-3 or SH. (a) Representative images of Evans blue stained extravasation 15 min after intraplantar injection of 0.5 mg/mL M-3, 0.5 mg/mL SH, or 10 μg/mL C48/80 in the left paw, or saline in the right paw. (b) Quantification of paw thickness increase after 15 min (c) Quantification of Evans blue leakage into the paw after 15 min. **(B)** Telangiectasia induced by M-3, SH. (H&E staining analysis; arrows indicated skin microvessels). **(C)** M-3 and SH induced MCs degranulation (Toluidine blue staining analysis; arrows indicated MCs).

In order to assess the role of M-3 and SH in the formation of pseudo-allergic reactions, we adopted a passive skin allergic model in mice. After intravenous injection, M-3 or SH were injected subcutaneously for 15 min. C48/80 was used as positive control. The pseudo allergic reaction was characterized by hind paw edema and Evans blue stain leakage into the paw. Subcutaneous saline injection (vehicle) did not cause inflammation of the hind paws (swelling and extravasation). On the contrary, M-3 or SH could induce extensive extravasation and swelling in mice. In a previous study, SH-induced pseudo-anaphylactic reactions associated with Mrgprb2 expression in mast cells (Liu et al., 2017). Therefore, we examined whether the development of hind paw inflammation following M-3 injection was related to mast cells, using Kit^*W-sh/W−sh*^ mice. In C57BL/6-Kit^*W-sh/W−sh*^ mice, M-3 induced hindpaw inflammation was nearly absent, suggesting that M-3 was related to mast cells. Next, MrgprB2-CKO mice were used to examine the relationship between the inflammation of the hind paw and the Mrgprb2 expression in the mast cells. Compared with the wild-type Mrgprb2 mice, the Mrgprb2-CKO mice showed virtually no hind paw inflammation (Figure 5A). In addition, M-3 induced telangiectasis in C57BL/6 mice (Figure 5B) and mast cell degranulation (Figure 5C). These effects, however, were practically absent in the Kit^*W-sh/W−sh*^ and Mrgprb2-CKO mice. These results indicate that the activation of mast cell mediators and subsequent vasodilation induced by M 3 may be associated with Mrgprb2, similar to that of SH.

### 3.8 Differences in the activation of MRGPRX2-mediated expression of inflammatory mediators in LAD2 cells *in vitro* by M-3, SH, and PAMP-12

**FIGURE 6:**
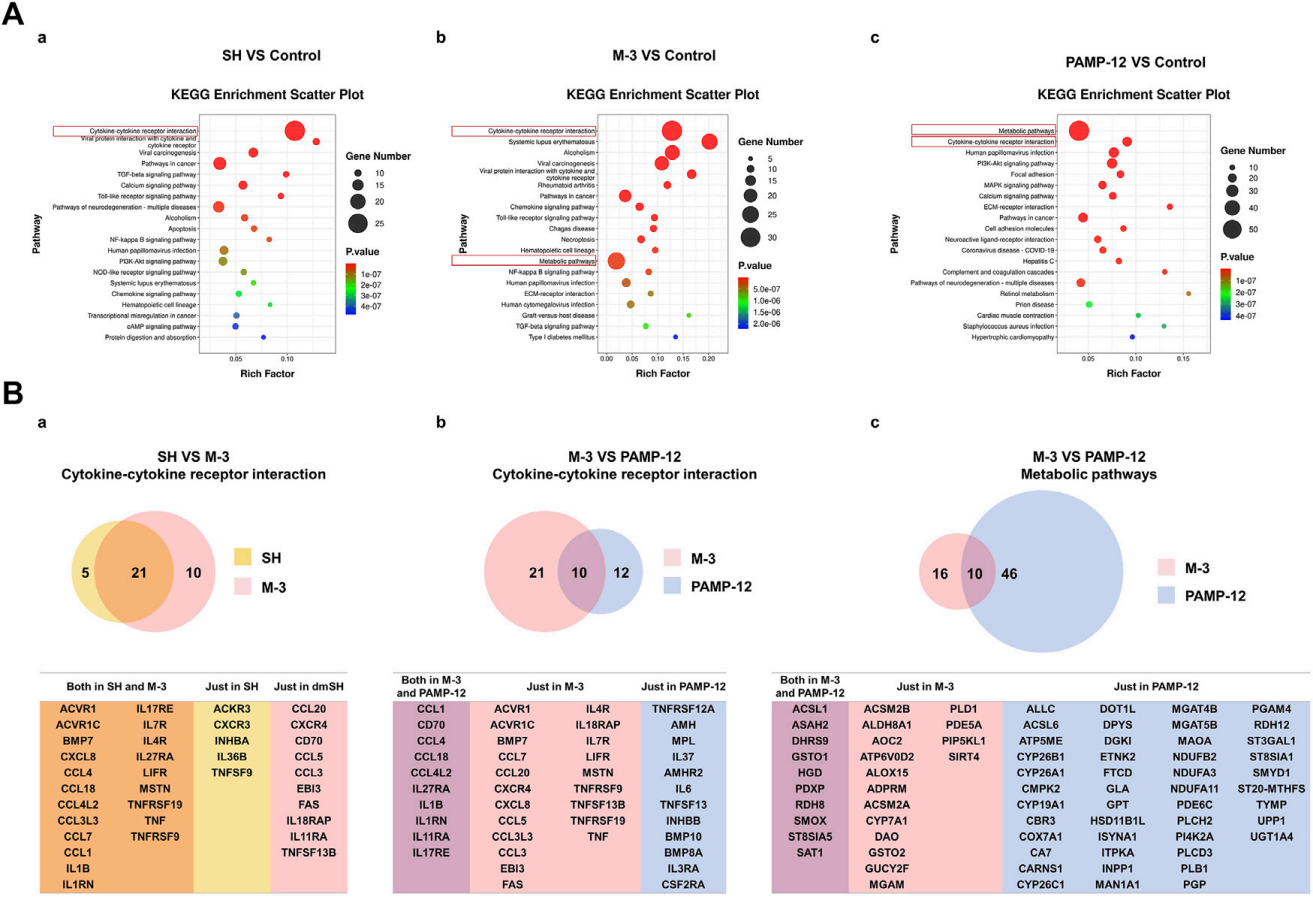
The mRNA sequencing analysis of SH, M-3, and PAMP-12 activated LAD2 cells. **(A)** KEGG pathway enrichment analysis of differentially expressed genes. 10 μM SH (a), 10 μM M-3 (b) and 2 μM PAMP-12 (c) Differentially expressed genes in LAD2 cells after 30 min treatment. **(B)** Wayne plots of differentially expressed genes. (a) Genes enriched in the SH and M-3 Cytokine-cytokine receptor interaction pathway; (b) Genes enriched in the M-3 and PAMP-12 Cytokine-cytokine receptor interaction pathway; (c) Genes enriched in the M-3 and PAMP 12 Metabolic pathway; (c) genes enriched in the M-3 and PAMP-12 Metabolic pathway.

PAMP (9-20) (PAMP-12) is a major product of the precursors of adrenomedullin. PAMP-12 can induce MRGPRX2-mediated mast cell degranulation reactions (Che et al., 2021). To investigate whether exogenous small-molecule compounds (M-3 and SH) and endogenous short peptides (PAMP-12) stimulate MRGPRX2 at different sites of action, leading to different effects, we analyzed the differentially expressed genes using RNA-sequencing (RNA-seq) *in vitro* after treatment of LAD2 cells with M-3, SH, and PAMP-12. Kyoto Encyclopedia and Genes and Genomes pathway enrichment analysis showed that M-3 and SH enriched the highest number of genes in the cytokine-cytokine receptor interaction pathway, whereas PAMP-12 enriched the highest number of genes in metabolic pathways. In addition, M-3 had a higher number of enriched genes in metabolic pathways and PAMP-12 had a higher number of enriched genes in the cytokine-cytokine receptor interaction pathway (Figure 6A). Next, we analyzed the major RNA-seq data for these pathways to identify genes that were differentially expressed (Figure 6B). Genes co-regulated by M-3 and SH in the cytokine-cytokine receptor interaction pathway were concentrated in the chemokine CC subfamily (CCL4, CCL18, CCL4L2, CCL3L3, CCL7, and CCL1) as well as the interleukin family, which invovles IL1B, IL1RN, IL17RE, IL7R, IL4R, and IL27RA (Figure 6B-a). Genes co-regulated by M-3 and PAMP-12 in the cytokine-cytokine receptor interaction pathway were similarly concentrated in the chemokine CC subfamily (CCL1, CCL4, CCL18, and CCL4L2) and the interleukin family (IL27RA, IL1B, IL1RN, IL11RA, and IL17RE) (Figure 6B-b). Unlike the M-3 and SH small-molecule compounds, the genes regulated by PAMP-12 were mainly involved in metabolic pathways (Figure 6B-c).

### 3.9 MRGPRX2 mediates M-3 triggered β-hexosaminidase release during anaphylaxis via activation of inositol 1,4,5-trisphosphate receptor (IP3R), phospholipase C γ (PLC-γ) and P38

**FIGURE 7:**
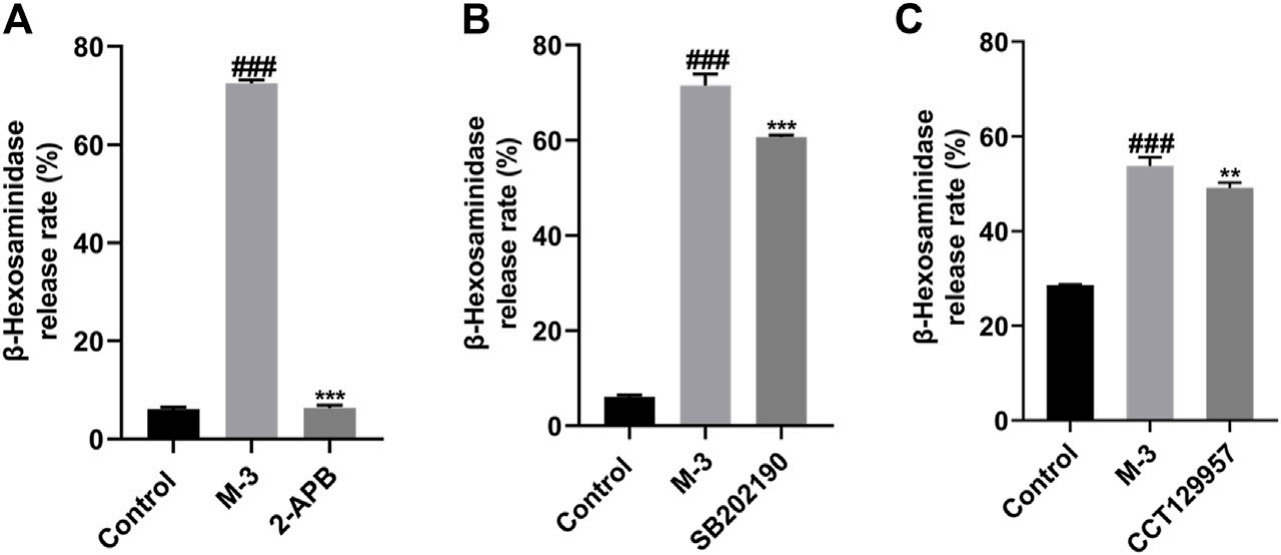
The β-hexosaminidase release from LAD2 cells by M-3 (50 μM) after the incubation of several antagonists. **(A)** IP3R inhibitor (2-APB), inhibitor concentration 45 μM; **(B)** P38 inhibitor (SB202190), inhibitor concentration 10 μM; **(C)** PLC-γ inhibitor (CCT129957), inhibitor concentration 3 μM. ^###^ *p* < 0.001 vs. Control group, **p < 0.01, ***p < 0.001 vs. M-3 group.

Phospholipase C gamma breaks down phosphatidylinositol-4,5-bisphosphate to form dissolved IP3. The release of calcium ions is triggered by the interaction between IP3 and IP3R, forming the “first wave”. This release is transient from endoplasmic reticulum storage. After pre-incubation of LAD2 cells with the IP3R inhibitor, the release of β-hexosaminidase from LAD2 cells triggered by M-3 was blocked (Figure 7A). In addition, we determined the level of β-hexosaminidase release after preincubation of LAD2 cells with PLC-γ and P38 inhibitors, and it was found that PLC-γ and P38 inhibitors similarly inhibited M-3-induced β-hexosaminidase release (Figures 7B, C).

## 4 Discussion

After entering the body, drugs are absorbed and metabolized in various ways to exert their pharmacological effects. A small number of drugs are metabolized to produce substances that still have pharmacological effects and may play the same role as prototype drugs. For example, the potent antitumor activity of irinotecan is associated with the rapid formation of its active metabolite SN-38 *in vivo* (Mullangi et al., 2010). Piperine and its metabolites are potential therapeutic agents that can interfere with the progression of various diseases, including chronic inflammation, cardiac and liver diseases, neurodegenerative diseases, and cancers, and have particularly important effects on the central nervous system (Azam et al., 2022). In addition, the clinical drug salazosulfapyridine used for the treatment of inflammatory bowel disease is a chemical combination of sulfapyridine and mesalazine (5-aminosalicylic acid; 5-ASA) (Kolassa et al., 1985), which is metabolized *in vivo* to produce sulfapyridine and mesalazine (Williams and Hallett, 1989; Böhm and Kruis, 2014), which have pharmacological effects in the treatment of ulcerative colitis, a type of inflammatory bowel disease (Böhm and Kruis, 2014; Mikami et al., 2023). In this study, we synthesized M-3, a metabolite of SH, and observed that M-3 possesses anti-inflammatory effects (Li et al., 2020; Zhou et al., 2021), elicits allergy-like reactions, and acts synergistically with SH. Upon entry into the body, SH is catalyzed by CYP2C9, CYP3A4, and CYP2D6 to expose the N atom and generate the active metabolite M-3 (Yao et al., 2007). These findings suggest that the structure may play an important role in the pharmacological action of the drug.

Mast cells are differentiated from hematopoietic stem cells and play important roles in allergic and inflammatory responses (Jackson et al., 2021). A large number of stimuli that can directly trigger mast cell degranulation and cause selective release of mediators, such as IgG, complement components, Toll-like receptor ligands, neuropeptides, cytokines, chemokines, and other inflammatory products (Yu et al., 2016). Multiple receptors on the surface of mast cells, such as IgE (FcεRI), IgG (FcγR), stem cell factor (KIT receptor or CD117), complement (including C5aR), and cytokines, activate a variety of signaling pathways upon stimulation (Komi et al., 2020). In addition, mast cells in the skin express MRGPRX2, which mediates diseases, such as rosacea, atopic dermatitis, nonhistaminergic itch, and pseudoallergy (Roy et al., 2021) and MRGPRX2 is involved in the majority of peptide stimulation-associated activation in human mast cells (Lu et al., 2017). SH triggers drug-like allergic reactions while treating rheumatoid arthritis and triggers allergic reactions via MRGPRX2-mediated mast cell activation. Therefore, M-3 may have the same effect on mast cells. However, little is known regarding the ability of M-3 to trigger drug-like allergy and its molecular mechanism of action. In this study, M-3 caused MRGPRX2-mediated release of β-hexosaminidase from mast cells, and IP3R inhibitors were able to inhibit the release of β-hexosaminidase, consistent with the fact that a few drugs lead to the release of calcium ions from the endoplasmic reticulum by promoting diacylglycerol and IP3 synthesis (Hoth and Penner, 1992; Putney and Mckay, 1999). In addition, our previous study found that artemisinic acid could block MRGPRX2-mediated mast cell activation through the Lyn-PLC-p38/ERK1/2/AKT-NF-κB signaling pathway (Ding et al., 2022), so we analyzed mast cell activation by inhibitors and found that β-hexosaminidase release was reduced after PLC-γ and P38 were inhibited, suggesting that M- 3 could activate MRGPRX2-mediated mast cell activation via PLC-γ/P38.

Rheumatoid arthritis is a systemic chronic inflammatory disease whose pathogenesis is unclear. Mast cells can act as a cellular link between many components of inflammatory arthritis (Lee et al., 2002). In osteoarthritis, mast cells are the only immune cell type that are found in high concentrations (Loucks et al., 2023). In addition, cytokines and proteases produced by mast cells are involved in the pathogenic process of rheumatoid arthritis, especially TNF, IL-1β, IL-17, and trypsin-like enzymes (Hueber et al., 2010; Xu and Chen, 2015). Elevated adenosine deaminase has been observed in patients with rheumatoid arthritis (Hamada et al., 2010; Brandao et al., 2023), and the enzyme can be cleaved by mast cell-released CPA3 to produce PAMP-12 (Brandao et al., 2023). Therefore, we comparatively analyzed the differences in mast cell activation induced by PAMP-12 and small-molecule drugs. Our findings demonstrate that SH and its active metabolite M-3 co-regulate the interleukin family and TNF, whereas the endogenous ligand PAMP-12 regulates interleukin family genes and metabolism-related pathway genes. This discrepancy suggests that the allergy-like effects triggered by M-3 and SH may simultaneously exacerbate the progression of arthritis, thereby reducing the therapeutic efficacy of these drugs.

In conclusion, this study demonstrates that M-3 induces anaphylactoid responses by activating mast cell MRGPRX2 for degranulation, resulting in the release of mediators, such as β-hexosaminidase, histamine, and TNF-α, and chemokines, such as MCP-1 and IL-8. M-3, as an active metabolite of SH, inhibits the release of β-hexosaminidase through signaling pathways, such as IP3R and PKC, P38, P2Y1, and GRK2 and other signaling pathways to inhibit the release of mast cell β-hexosaminidase, which is associated with mast cell activation. The findings confirm the role and potential molecular mechanisms of M-3-induced anaphylaxis, and provide new guidance for the study of drug-induced anaphylaxis.

## Data Availability

The data that support the findings of this study are openly available at Baidu cloud database [https://pan.baidu.com/s/1k3oFNf2tZkpFOInOMO7BNQ], extract code [6M26].
